# Addressing the Horizontal Gender Division of Labor: A Case Study of Support and Obstacles in a Heavy Industry Plant in Iceland

**DOI:** 10.1007/s11199-018-0915-7

**Published:** 2018-03-29

**Authors:** Gudbjörg Linda Rafnsdóttir, Jill Weigt

**Affiliations:** 1 Department of Sociology, University of Iceland, 101 Reykjavík, Iceland; 2 Department of Sociology, California State University, San Marcos, USA

**Keywords:** Gender equality, Gender segregation, Professional role, Stereotyped behavior, Workplace intervention, Workplace policy

## Abstract

In the present article, we analyze a project in a heavy industry plant in Iceland in which the management aims to hire an equal number of women and men and, thereby, to work against the gender segregation of work. For their efforts, called the 50/50 strategy, the plant has received national and international awards. Observations and semi-structured interviews were conducted during five visits to the plant, including 85 interviews with 72 individuals, 49 women and 23 men. We found extensive support for the policy. The managers saw business opportunities in it, but although the employees supported the policy because it was seen as fair and modern, they doubted that achieving equal gender representation would be possible. The main emphasis so far has been on designing work organization and equipment, advertising the policy, presenting job opportunities to women outside the plant, and encouraging both genders to acquire the “right” education. Why the 50/50 target has not been reached lies partly in gender stereotypes outside the plant. Furthermore, our findings suggest that the next steps should be to challenge an alleged male working-culture within the plant. If they fail to do so, their efforts to eliminate horizontal gender segregation are unlikely to succeed and may even become counterproductive.

In economically developed countries, equal opportunities for women and men in the labor market are seen as socially desirable and pragmatically sound whereas gender segregation is seen as problematic. A number of scholars show that when diversity is increased and both women and men are present and integrated, corporations perform better (Adams and Ferreira 2009; European Parliament 2011; Matsa and Miller 2013; McKinsey and Company 2010; Teigen 2011; Wang and Kelan 2013). As Ellingsæter (2013) and Teigen (2011) show, other scholars see gender diversity more as a matter of justice and equality. Consequently, various actions and initiatives have been taken in many countries, especially since the 1970s, to reduce occupational gender segregation (Browne 2006), even if it has also been criticized (e.g., Hakim 2007, 2008). Despite different actions to decrease or eliminate gendered occupational segregation, it still persists (Browne 2006; Pascal 2012). Gender segregation is so deeply embedded in workplace norms and employment practices that gender segregation would still require between 25% and 40% or more of men or women to change occupations to achieve complete gender integration (Cha 2013; Ellingsæter 2013). These estimates show that the gender system is deeply entwined with social hierarchy and gender stereotypes. These stereotypes contain beliefs that associate status and competence with men rather than with women (Ridgeway 2001).

In the present article, we analyze how horizontal gender segregation is kept in place in a heavy industry plant in Iceland, here called Metalicorp, which has aimed to hire an equal number of women and men to the plant and thereby eliminate overall gender segregation. The management team of this hazardous, heavy metal, multinational industry plant identified gender segregation as problematic and therefore committed to changing common workplace norms and practices to be able to attract women to the plant. Metalicorp has won both national and international awards for its gender equality policy and its aim to eliminate gender segregation. Closely examining their efforts, we will mainly discuss three issues: the elimination of the overall gender segregation of the plant, employees’ view of gender equality laws and policies, and the implementation of the plant’s aim to recruit an equal proportion of women and men to the plant.

Our data are unique, as this is, as far as we know, the only heavy metal industry plant with a mission statement committed not only to increasing the number of women employees, but also to hiring an equal proportion of women and men across divisions in the plant (hereafter often addressed as the 50/50 target or strategy). Consequently, the goal of the plant is to decrease significantly the overall gender division of labor in the plant. To give context as to what possibly hinders the plant’s policy, and to ground our theoretical approach, we now turn to the literature on the gendered division of labor.

## Gendered Division of Labor

In recent decades, the characteristics of the gendered division of labor in the workplace have been mapped and their causes examined. However, numerous studies show that whereas women have entered white-collar, male-dominated professions and management positions, the flow of men into what has been seen as traditional “women’s” jobs and of women into male blue-collar jobs has been much slower (Cross and Bagilhole 2002; Ellingsæter 2013).

Several studies show that gender inequities persist even after organizations institute policies designed to reduce them, including affirmative action policies (Kalev et al. 2006), merit-based pay programs (Castilla 2008), problem-solving teams, and job training (Kalev 2009). Interestingly, comparative studies show that “gender traditional” countries like Greece, Italy, Japan, and Portugal tend to have lower overall levels of gender occupational segregation than do countries that are typically seen as relatively gender egalitarian, like the Nordic countries (Browne 2006; Charles 2003; Charles and Grusky 2005; Halldén 2014; Jarman et al. 2012). The reason is that where most women participate in the labor market (like in the Nordic countries), the service sector is large and women are most likely to take up new jobs that are created, particularly in the health and education sectors (Halldén 2014). Nevertheless, the Nordic countries score high on the European Gender Equality Index, both in relation to gender equality generally and at work (European Institute for Gender Equality 2013), as well as on the gender wage gap index, where Iceland has been ranked number one since 2009 (World Economic Forum 2017).

Gendered occupational roles result in horizontal and vertical segregation. *Horizontal segregation*—the main focus of our article—refers to differences in the gender breakdown present across occupations, whereas the term *vertical segregation* describes men’s domination of the highest-status jobs in both traditionally male and traditionally female occupations (Browne 2006; Charles 2003; Charles and Grusky 2005). In an attempt to explain why vertical and horizontal occupational segregation persists, the “More Women in Senior Positions” (European Commission 2010) report points out two groups of gender-based stereotypes that pigeonhole women and men into different occupational roles and sectors of employment. The first and most fundamental is the stereotype that separates the responsibilities of providing and caring for the family between men and women and leaves women in the home or perpetuates the view that women should take primary responsibility for raising the family. This stereotype raises doubts about women’s capacity to fulfill this domestic role together with a professional career. The second group of stereotypes relates to gender-based personal characteristics, prompting women and men to choose different jobs. These stereotypes presume that women and men are essentially different, have different skills and abilities, and as such are best suited to different types of jobs.

The overall segregation level is not, however, necessarily seen by scholars a matter of gender inequality. Thus, the fact that the Nordic countries score high on the Gender Gap Index (World Economic Forum 2017) at the same time as the Nordic labor market remains highly gender segregated is seen by some scholars as demonstrating that gender essentialism, which encourages segregation, and gender egalitarianism can operate simultaneously (Charles and Grusky 2005; Ellingsæter 2013; Hakim 2007; Jarman et al. 2012). We will address this point in our article. Nevertheless, this pattern is generally seen as problematic because gender segregation can affect earnings, career mobility, and work autonomy for the individual; can reinforce cultural gender stereotypes in society and create a less-flexible labor market (Ellingsæter 2013; Roseberry and Roos 2014).

Even though the gender segregation of work is a global phenomenon, it varies widely across regions and thus, according to some scholars, there is no prerequisite for talking about worldwide gender segregation because the mix of vertical and horizontal mechanisms is very diverse (Anker 1998; Charles and Grusky 2005; Jarman et al. 2012). However, several studies on employees who work in jobs seen as traditional for the other gender in their regions show that these employees often meet hindrances (Heilman and Okimoto 2007; Røthing 2006; Warming 2013). Various studies show that women, rather than men, are still expected to behave collectively and demonstrate concern for others through “feminine” attributes such as kindness, sympathy, and understanding instead of demonstrating dominance and competitiveness, behaviors typically prescribed for men (Heilman and Okimoto 2007), and that these role proscriptions hinder the elimination of a gendered labor market (Heilman 2001). Heilman and Okimoto (2007) even showing that women are penalized for success in “male tasks.” Garcia-Retamero and López-Zafra (2006) demonstrated incidences of prejudice against women working in industries incongruent with their gender role. Similarly, Røthing (2006) showed that women and men who cross the gender bridge in the workplace are socially marginalized and feel they need to work on opposite-gender terms and outperform expectations in order to be regarded as equal to their counterparts. Despite this marginalization, Ely and Meyerson’s (2000) study on offshore oil platforms in the Gulf of Mexico shows that it is possible in a very masculine work environment to “undo gender” when the structures that promote gendered behavior are shown to threaten occupational safety. By making the employees (all men) more aware of safety measures, the men behaved in what was seen as counter-stereotypical ways, readily acknowledging their physical limitations, publicly admitting their mistakes, and openly attending to their own and others’ feelings when interacting with each other. However, studies of men working in female-dominated organizations in Denmark (Warming 2013) and Norway (Røthing 2006) also show that although these men are ready to undo gender by working in traditional female jobs, they face similar prejudices as women working in male-dominated organizations in that they complain about being professionally and socially marginalized. Their attitude mirrors that of women in male-dominated work environments by complaining about “female work culture” and women’s perceived interest in dominating the workplace.

Roseberry and Roos (2014) argued that the discrimination narrative does not direct our attention to how people act in workplaces where, without any malicious intent, they do and say things that create gender barriers. Although we agree with Roseberry and Roos in this respect, our study aims to shed light on factors that keep horizontal gender segregation in place in Metalicorp, the plant we are studying. First, however, we will discuss the political context of the plant because we believe that such context is crucial to Metalicorp’s project and our arguments.

## Context of the Present Study at Metalicorp

Political and societal context is an important factor in determining whether a gender policy is to succeed. Gender equality acts have certainly been implemented in many countries, and because organizations are potent sites for reproducing traditional gender roles (Ely and Meyerson 2000), some of these acts address gender discrimination within companies. Promotion of gender equality has also become part of the core values of many companies (Walby 2011). That is also the case for Metalicorp. It operates under Iceland’s gender equality act, which states that all individuals “shall have equal opportunities to benefit from their own enterprise and to develop their skills irrespective of gender” (Act on Equal Status 2008, Article 1). The first article of this act mentions 10 ways to reach gender equality, including “changing traditional gender images and working against negative stereotypes regarding the roles of women and men.” The gender equality act touches on horizontal gender segregation by stating that “Employers shall work specifically to put women and men on an equal footing within their enterprise or institution and to take steps to avoid jobs being classified as specifically women’s or men’s jobs” (Article 18). The gender equality act also requires that every workplace with more than 25 employees develop their own gender equality policy. The act states specifically that each policy shall include a statement of aims along with a clear plan of how those aims are to be achieved.

Even though Metalicorp’s equal opportunity policy is based on the Icelandic Gender Equality Act no. 10/2008, Metalicorp’s workplace policy surpasses the act’s expectations by stating that the plant “aims for 50% of the company’s employees to be women.” Metalicorp’s policy further states that:

All job positions within the company are open to both men and women and shall be designed in order that both genders are able to perform them in a safe way. Both parents are encouraged to harmonize their work with family life and shoulder equal responsibility for the care and upbringing of their children.

Thus, taking a stand with this policy, the plant aims to recruit an equal number of women and men in the plant, both in production and management. This is particularly ambitious given the fact that most of the jobs at the plant are thought of by the wider community as strongly masculine jobs, especially in the production site. The international corporation to which Metalicorp belongs aims to increase the number of women in middle-management positions (not just blue-collar jobs). Consequently, the top management team of the corporation receives bonuses based on the percentage of women in these middle-management positions.

According to a societal report for 2016, Metalicorp employed 554 people directly that year, 136 (24.5%) of whom were women, which is much lower than the target of 50/50. Most of the employees worked within manufacturing—244 men and 85 (25.8%) women. The manufacturing side employs mainly unskilled people. Craftspeople consisted of 74 men and 7 (8.6%) women. The executive board consisted of 7 men and 2 (22.2%) women, while the so-called experts and managers were 100 men and 44 (30.5%) women. Thus, the challenge is largest within manufacturing, where the working environment is also hardest in relation to danger, dirt, and physical strength, whereas the group experts and managers comes closest to the 50/50 target. Metalicorp also employs 350 people indirectly (via contractors) onsite. These employees are not part of our study.

In addition to the 50/50 target, the plant aims to eliminate any gender pay gap and ensure equal promotion for women and men. As a part of that target, the status of the wages among women and men is published regularly, and is based on eight job categories. According to Metalicorp’s societal report for 2016, female managers made 98% of males’ salary; female experts, 96%; female engineers, 79%; female technically skilled and specialized workers, 89%; female office workers, 100%; female retailers, 96%; female craftspeople, 90%; and female mechanical personnel, 92%. Thus, in 2016, a gender pay gap existed to some extent. The report states that the pay gap is because men in general have worked for more years at the plant than women have and thus men have earned higher salaries because of seniority.

The plant also addresses generous policies regarding work–life balance, parental leave, and family responsibility, as well as protections against sexual harassment. As a reaction to the #MeToo campaign (a global movement on social media to address sexual harassment), the plant is aiming to strengthen the focus on the working culture and launch a project on harassment and male culture in general, a culture that might work against women and the 50/50 target.

When the plant’s construction was on the drawing board, it was criticized for becoming a “big man place” in a community that needed new jobs for women as well as for men. The plant met this criticism by committing to create a plant that was “woman friendly” and that was constructed to allow men and women to perform all tasks in the plant equally. This is important, because as Blair-Loy et al. (2011) pointed out, corporate values or mission statements (sometimes called corporate responsibility or guiding principles) are crucial in the management of public image and identity. Mission statements in particular “outline where a firm is headed; how it plans to get there; what its priorities, values, and beliefs are; and how it is distinctive” (p. 429). Through its equal opportunity policy, Metalicorp publically constructed itself as a workplace committed to gender equality above and beyond already progressive Icelandic law. This is part of the company’s larger vision and plays an important role in constructing their public image.

In an interview with the director of the plant with one of the main radio stations in Iceland in December 2017, he said that the plant has not given up its target to hire an equal number of women and men and that next on the agenda was to analyze the workplace culture. Specifically he said:

I think the key to creating a workplace that welcomes a wide range of staff is through the workplace culture. We can say that it is important that women, for example, do not perceive themselves as a guest at a male-oriented workplace. I think we need to address what is appropriate or improper behavior or appearance at the workplace. As an example, the man who unexpected takes the tool of the woman because he thinks he can do it better. He thinks he is polite, but this is not a good workplace culture.

To raise the plant’s gender egalitarian image, newspaper articles have been written through the years and advertisements published to introduce the company’s policy to the region. The policy states that, “…if there is an unequal representation of women and men, the underrepresented gender shall be encouraged to apply for employment.” In accordance with this stipulation, special public meetings have been held for women in an effort to present the plant as women- and family-friendly. Also, the redesigns of equipment to be more ergonomically appropriate for both men and women have been highlighted so that both genders can perform the work safely.

Nevertheless, we realized when observing the plant that the production side of the plant involves heavy machinery so that the work is quite physically and mentally demanding. In some units, magnetic fields are so intense that pregnant women are prohibited from working. Chemicals often permeate the air. The temperature is sometimes high, not the least for employees who also must wear overalls, thick boots, helmets, glasses, and ear props for safety. The employees describe themselves as sweaty and dirty. The working area is large and requires walking long distances. Some of the tasks are mentally demanding and even dangerous, but to some they also feel “empowering.” The work entails transporting material weighing many tons, transferring heavy metal plates via cranes, and dealing with boiling metals. The work is prearranged by “shift groups,” each comprising around 30 employees, and shift supervisors also help to determine the division of labor and how often employees rotate between tasks. Until recently, all shifts were 12 h long.

It quickly became obvious that it was not an easy task to eliminate horizontal gender segregation in the company because the working environment was traditionally seen as highly masculine. The percentage of women was at its highest when the plant opened in 2007, accounting for 33% of the workforce. As far as we are aware, no similar plant in the world has had such a high percentage of female employees. Since then, however, the number of women has decreased, down to 21% by 2013. It has actually increased again, even as the 12-h shift were reduced to 8-h shifts. At the end of 2017, the proportion of women overall was around 25%.

## The Icelandic Setting

Metalicorp’s placement in Iceland is key because it is a country with a generous welfare state and shares with the other Nordic countries a progressive approach to gender equality. The “Nordic model” is known as an active welfare regime, promoting “defamilization” that has been defined as “the degree to which individual adults can uphold a socially acceptable standard of living, independently of family relationships, either through paid work or through social security provisions” (Lister 1997, p. 173; see also Bambra 2004; Eydal and Friðriksdóttir 2012). Thus, Iceland has moved away from the male breadwinner model (Pascal 2012) by supporting a range of policies to strengthen women’s labor market position and the reconciliation of work and family life. Hence, up to a certain limit, parents receive 80% paid parental leave for a total of 9 months. Fathers and mothers are each given 3 months, plus a further 3 months to share as they see fit (Gender Equality in Iceland n.d.; Gíslason 2007). There are inexpensive municipal childcare facilities for pre-school children and a legal right for parents to return to their jobs after childbirth and to stay home if their child is sick.

Women in Iceland have the highest labor force participation rates among OECD countries: 86.9% for 25–64-year-old women in 2016 (OECD 2018), they form 47% of the labor force, and work on average 35 h a week compared to 44 h for men (Statistics Iceland 2018). According to the World Economic Forum’s Global Gender Gap Index, the country has been ranked number one for gender equality in the world since 2009 (World Economic Forum 2017). The Index benchmarks national gender gaps based on economic-, political-, education-, and health-based criteria. Iceland is the second country in Europe and in the world, after Norway, to implement 40% gender quotas on boards of corporations and in pension funds (Act on Boards of Directors 2010; Act Boards of Pension Funds 2011; Rafnsdóttir et al. 2014). The laws were enacted in September 2013. In 2008, the Icelandic government introduced gender quotas on government committees, councils, and boards (Act on Equal Status 2008). In addition, Iceland was the first country in the world to implement the equal pay standard mentioned previously. The latest government action is a development of an equal pay management system, called the Equal Pay Standard, that will help employers to prevent salary discrimination and enable them to become certified. The Equal Pay Standard was mandated for companies and institutions with 25 employees or more, with amendments to the Act on Equal Status and Equal Rights of Women and Men (2008; European Commission 2017).

In light of this background, it is clear that the political context in Iceland should not hinder Metalicorp from reaching its target of hiring an equal number of women and men to the plant. Challenging gender segregation has instead benefitted the plant by helping Metalicorp to foster a positive public image, allowing the company to present themselves as responsible employers who are responsive to the needs of the community and to brand themselves as consistent with Icelandic values. However, balancing gender representation in the company has proven to be a difficult task. In the present article, we aim to develop an understanding of why this is the case. We agree with Browne (2006), who claimed that more research is needed about the current mechanisms causing horizontal gender segregation and the ineffectiveness of current legal and policy solutions. Our study aims to fill this academic research gap and at the same time increase practical knowledge of the matter. We see it as especially important to understand the mismatch Brown observes between legal and policy approaches and the reality employees face in their everyday lives. Based on our data, we analyze the factors facilitating the policy as well as obstacles to its implementation. By doing so, we shed light on factors that keep gender segregation in place and prevent equal representation of women and men at the plant, despite Metalicorp’s gender equality policy and core values and the favorable political and societal context in Iceland.

## Method

### Participants and Procedure

The approach in our study is qualitative, and the empirical data are mainly based on interviews and observations conducted over five visits to Metalicorp, including one in 2010, two in 2011, one in 2012, and one 2013. In every visit, we also observed the plant in general, and we were accompanied by managers or middle managers due to security issues. The main aim of the observations was to understand the working conditions at the plant and to contextualize the interviews.

During each visit, we stayed on-site for up to a week. We completed 85 semi-structured interviews with 72 individuals: 49 women and 23 men, 68 of whom are Icelandic. A majority, 61 individuals, was employed at the plant, with 39 working in production and 22 in administration or management. In addition, we interviewed 7 former women employees and 11 additional individuals (5 women and 6 men) who had a connection to the plant through their positions in the community, such as through the local municipal government, local labor unions, or other related projects. Initially, because we had theoretical and substantive interests in women’s experiences, we focused on women who were presently or formerly employed at the plant. During each trip, we made a point of re-interviewing upper management and other key informants about the implementation of the gender equality policy. For updating our facts and figures when writing the article, we interviewed two of the managers in 2017 and 2018.

Interviewees were sampled primarily through snowball sampling. Thus, we conducted interviews with those recommended to us by previous interviewees or those who for other reasons were of interest to the project, such as municipal officials who could speak to the role of the plant in the community. Only one potential interviewee rejected our request because she was on parental leave with a newborn baby and lacking time.

Our objective, from the start, has been to document the process of implementing the gender equality policy over time rather than to simply compare and make claims about the degree of change occurring between each visit to the site. As such, our approach to the study has been ethnographic and organic, focusing on particular issues as they arise during each visit rather than maintaining a strictly longitudinal approach. We typically reviewed previous transcripts before each visit and, when necessary, referred back to them with repeat or subsequent interviews. With regard to employees, interview questions touched on their work at the plant, their experiences of being a man or a woman working at the plant, their opinions on the gender equality plan and its possible strengths and weaknesses, the reason why it was adopted, and why they think female participation in the workforce has dropped since the plant opened in 2007.

Interviews with community members also focused on the gender equality efforts of the plant. The interviews, which lasted between 30 and 120 min each, were tape-recorded and transcribed verbatim by a professional company, except for a few instances in which they were conducted in situ during observations. Notes were recorded on the spot in these instances. Although the interviews were semi-structured, based on a list of predetermined questions that focused on perspectives and experiences with the gender equality policy, work experiences at the plant, and work–family balance, participants were encouraged to explore issues they felt were important. Thus, the interviews took the form of a dialogue in which the interviewees and their interpretations guided the conversations. Interviewees routinely answered all of our questions, except for a few who asked us to turn off the tape while discussing difficult personal matters, conflict with coworkers, or difficult emotions such as anger, sadness, and frustration. Two former employees were unwilling to discuss the plant’s policy and negative experiences they had working at the plant. In these instances, notes were taken. We also took notes during and after the interviews and during our observations; we compared and frequently discussed them. Throughout our visits, we discussed the interviews and wrote and compared analytical and observational notes at the end of each day.

Both authors were present for all interviews conducted in English. Although most of the interviewees were fluent in English, which was not their mother tongue, a few felt impeded and were not able to fully express their perspectives and experiences as they wanted. A fluent Icelandic speaker (the first author) was present in all of the interviews to provide translation as needed. Three interviews were conducted in Icelandic because the interviewees did not speak English.

Metalicorp’s management team supported the study, which gave employees the option of being interviewed at work or nearby in the conference room of the researchers’ hotel. Most current employees chose to be interviewed at work. Former employees and three current employees who still lived close to the plant chose to be interviewed at the hotel. Three interviewees opted to be interviewed in their homes. Four former employees were interviewed at the first author’s offices. The interviews with the community members were conducted in their own offices. Our research project was reviewed by the institutional review board at the second author’s home institution and was approved for the protection of human subjects. Each interviewee was informed about the objectives and risks of the research and was assured that their confidentiality would be protected.

### Analysis

We approached our research using constructivist grounded theory (Charmaz 2006), meaning that our theory development is grounded in our interview data. Our aim was to understand interviewees’ “experience(s) within embedded, hidden networks, situations, and relationships, and making visible hierarchies of power, communication, and opportunity” (Creswell 2009, p. 65). Our work follows from Burr (1995, p. 7) who argues that it is only “when people talk to each other, the world gets constructed.” In that spirit, we draw on a social constructionist stance, arguing that there is no single truth to discover but that multiple realities are experienced by each of us subjectively (Marsh and Musson 2007; Veale and Gold 1998). In line with Marsh and Musson (2007), we stress that the performances of the employees’ identity we display here are ultimately the products of the participants’ interactions with us in the context of the interviews. As such, the analysis we offer here is the product of the systems of meanings that are created and negotiated between the researchers and the interviewees in the specific situations of the interviews.

All interview data were coded, bearing in mind our substantive interests in the ways the gender equality policy was carried out and its subsequent outcomes. We used Atlas.ti to manage our data and to highlight quotes from individual interviews. Using an interpretive approach, we coded the interviews and allowed the themes to emerge from the data (Corbin and Strauss 1990). Nevertheless, we asked questions of the data and refined our coding categories throughout the analysis. Themes were developed through the interplay of our theoretical and substantive interests and what the data revealed.

To maintain interviewees’ confidentiality, and because Iceland has such a small population, we obscured identifying characteristics more than we might if we were conducting research in a larger community. Thus, the only demographics we cite are gender; whether they are currently, formerly, or never employed at the plant; and whether interviewees were employees in production or management/administration.

## Results

The employees we interviewed held contradictory attitudes toward the gender equality policy at the plant. In fact, some of the interviewees simultaneously supported the policy and ignored it. Nevertheless, almost none were against the policy. The reason why they supported the policy was not necessarily that they believed in the same jobs for both genders. Rather, they just liked the aura of modernity they thought the gender equality policy itself brought to the plant, regardless of whether the policy would actually be fully carried out. Yet, the local setting is also important—a small town in need of jobs for both genders. The interviewed managers were committed to the aim of hiring an equal number of women and men to the plant and, as claimed by one of the top leaders, “It is not only nice words. Any goal other than 50/50 is not acceptable because we live in [a] community [with] 50 percent women, 50 percent male. We will get there. But it will take years.” This quote demonstrates that achieving equal representation is a long-term goal and that they feel pressure from the local community to hire more women to the plant.

Despite the policy, the percentage of women working at the plant decreased and the turnover rate was high, up to 30% for both men and women. One of the managers pointed out that when they analyzed the turnover rate 3 years after the startup of the plant, they found, to their surprise, that men were just as likely to leave the plant as women were. The percentage of women working at the plant decreased, not only because they quit, but also because, despite the policy, fewer women than men had been hired at the plant across divisions. This turnover rate is partly because young people do not like to settle down for many years in the small community where the plant is located. When couples move to the community to work at the plant, women are usually the first to move back to larger communities after a year or two, according to one of the managers, and husbands follow soon after. Many interviewees reported that the problem is not simply the work itself, but that most employees prefer to live in communities that offer more culturally than the one where the plant is located.

Nevertheless, a common reason the managers gave for the lower percentage of women was that too few women applied for positions and that they did not have the “right” education for the plant. As one manager stated, “We need more women educated as tradesmen, technicians, engineers. We try to open the young women’s and girls’ eyes on that.” The plant’s public relations department has tried to do just that. Special women’s days are held locally during which women working at the plant present job opportunities to other women in the community. In addition, employees visit the elementary and high schools in the area, educating children about the work of the plant and encouraging both boys and girls to acquire the “right” education for the plant. Nevertheless, it is important to keep in mind that many of the plants’ jobs do not require any special education, but simply job training.

### Support for the Policy

Though employees supported the policy, they did not believe it was necessarily achievable. Our analysis revealed three main reasons behind their support, in addition to the business perspective we mentioned previously: (a) the gender equality policy is seen as modern, (b) more women working at the plant can enhance the work atmosphere and morale, and (c) women are perceived as safer and more skillful workers than men are. Rather than representing deep commitment to gender equality, the last two reasons reveal essentialist understandings of gender. As a means to comprehend the persistence of gender segregation in this context, we examine each reason in greater depth to better understand the connection between support for the policy and disbelief that it can be achieved.

#### The Policy is Modern

Despite the decreased percentage of women working at the plant, managers at Metalicorp were generally positive about the gender equality policy. They saw the policy as decorative—as branding that would paint the plant as progressive and cutting-edge. In the words of one woman middle manager: “I think it is a good publicity thing, but I don’t really see the need for it.” Although the publicity garnered by the policy is helpful for the image of the plant, she does not see the gender division in work as problematic. The policy was, in her mind, more like a modern “blue-sky branding.” Nevertheless she was not opposed to the policy, even if she was a bit tired of ongoing discussions about women’s position at the plant. Many of the interviewees, both women and men, referred to Iceland’s number-one standing in the world stage in terms of gender equality and saw that as a reason for the plant’s policy: “This is the way we do things in Iceland.”

Upper management supported the policy, but in part for different reasons than the employees generally. One of the managers expressed the importance of the policy in this way:

Why is it [the 50/50 policy] important for us? …Honestly, it’s nothing but business. Call it philosophy, but it’s really business because we will only be allowed to be here as long as we have a good relationship with the community.

A good relationship with the community means that the plant needs to serve and employ both women and men. However, although they were aware of the policy, most of the interviewed employees did not believe that in practice the plant would ever reach the 50/50 target, at least not in the near future. Interestingly, this gap between support for and doubt about the achievability of the policy was not seen as problematic for the employees interviewed. One young man reported: “It is good at least to have it as a goal, even if it’s not realistic.” This man, and many other interviewees, saw the gender equality policy as positive, but utopian.

#### Women Enhance the Work Atmosphere

The atmosphere at the plant was described by interviewees as a traditional masculine working-class culture. However, one of the most frequent answers given when asked why the plant has implemented the 50/50 policy relates to enhancing the working atmosphere. “If anything, it’s…just good for the morale, I think,” reported one male operator. A woman in production claimed that when women and men work side-by-side, the employees talk together more and are happier. “I don’t know how to describe it…it is just more lively on the shift when genders are blended.” “Women beautify our life,” said one of the male operators who supported the policy but nevertheless perceived that the position of operator was too physically demanding for women. However, he told us that he liked having a few women on each shift. Many interviewees, especially men, asserted that women have more concern for others, that they are kinder and more understanding, and that men and women make better teams “because they are different.” For example, one male employee said, “The atmosphere becomes softer and more polite when women are around.” When asked why he supports the gender equality policy, another male operator said:

You have heard about when a man says, “My wife makes me a better man.” That is the concept. We make each other better because we have a different point of view. And most women are better in caretaking than men…It’s about stability and it’s about caretaking…So a team has to be mixed with both genders…so that is the reason.

Even though both men and women opined that increased gender balance improves the working atmosphere in the plant, men more frequently voiced essentialist ideas about the presumed differences between women and men. Women were more likely to say that gender did not matter in terms of job competence and that women and men could accomplish the same working tasks. And even if the employees talk about the benefits of gender integration, our observations show that the dining room was predominantly divided by gender.

#### Women are Safer and Better Operators

Another reason frequently cited by both men and women for supporting the 50/50 policy is that women are considered better and safer operators. Because they possess “built-in responsibility” and “take care of everything,” women are perceived not only as caring more about their coworkers, but also as better at doing some tasks. It is widely believed in the plant that women work better than men at tasks requiring precision and tolerance. One male employee claimed, for example,

Less error is made if you have women, because they often stop and think and ask…she knows the danger, she never takes any risks, she is always particular with the pre-flags (warnings), you know, there are all kind of things like that.

Referring to driving oversized trucks and operating cranes, another male employee said, “I have seen women better at things…concerning safety…They are more likely to stop and ask…making sure that they are doing the right things.” Making mistakes in this environment can have some serious consequences so safety is an important issue and another reason why women’s presence should be solicited, according to many interviewees. One man summed up this assumption by saying: “Women are [by] nature more careful…these are known facts.” Because women are assumed to “naturally” be risk-averse, more caring, more careful, and more responsible, employees supported the 50/50 policy. We again see here that their support for the policy rested on essentialist notions of difference rather than a commitment to gender equality per se.

### Hindrances in Practice

Even though the interviewees report that they support the gender equality policy in the plant, they identify clear hindrances to achieving the 50/50 objective, barriers they more or less believe will persist. The gender equality policy guarantees that at least a steady trickle of women will be present in the plant, but a gender-egalitarian plant without horizontal gender segregation is, despite general support for the policy, not of a special concern to most interviewees. Employees gave three main reasons for why they think the policy is unrealistic and ultimately will not succeed: (a) Traditional views on gender persist in the region, which will prevent adequate numbers of women from seeking employment at the plant; (b) 12-h shifts interfere with women’s domestic duties; and (c) jobs available at the plant involve too much physical strain for women. We examine each reason in greater depth to better understand the contradiction between support for the workplace policy and disbelief that it will be achieved.

#### Traditional Views on Gender and Work

Several interviewees told us directly that the main obstacle to reaching the 50/50 target rests in the stereotypical perspectives of potential and actual employees. As one of the male operators explained: “I have [a] lot of girlfriends, and they always say, like, ‘I would never, never in my life work that kind of job.’”

Beyond what we heard directly from interviewees, it was clear that traditional attitudes regarding an appropriate gender hierarchy persisted. A survey conducted by the plant’s gender equality committee shows that skepticism about the 50/50 target is strongest among young men employed at the plant. Some of our interviews helped to explain this skepticism. For instance, one woman who worked as a middle manager reported that she experienced some resistance to her authority from younger men:

The worst age is the thirty to forty. There is something in there, they are themselves trying to balance home, new babies, wife, the work, climbing up … and then big mama comes and tells them off. No, that’s not the deal (laugh).

Some employees were uncomfortable with the fact that women are sometimes hired over men to achieve gender balance. For example, one young male operator told us he supported the gender equality policy and the 50/50 target; it is “…a good idea, of course…because I just think that everyone should be equal.” A few minutes later, he relayed that he and a woman friend applied for the same job at another plant, but because that company was trying to improve its own gender balance, he was turned down. He explained his reaction:

It pissed me off. Yeah. Because I really needed a job. And it pissed me off because my friend got a job…I didn’t get the job but she did get the job. Just because she was a woman. I—yeah, I have to be honest. It pissed me off a lot.

He supported the gender equality policy, until the ideals behind it affected him adversely. A male administrator echoed the same sentiment: “Overall, I think people are pretty satisfied with the policy,” although he knows men who have “been pissed off” when they were not promoted. Similarly, a female technician, who also said she supported the policy, noted that other employees, both men and women, were tired of the extra attention the female employees received by working in a male-dominated area, and she agreed: “It is getting a little bit too much sometimes about these women.” She relayed that her male coworkers call for men’s days or men’s lunches, which are similar to women’s days and lunches that are used to call attention to and celebrate women working in the plant.

#### Shift Work Interferes with Women’s Domestic Duties

Most of the interviewees, but not necessarily the mothers themselves, said that the 12-h shifts at the plant work against women’s equal participation at the plant because they are not family-friendly, especially for mothers of small children. One male operator explained: “Usually the women spend more time with the children, so 12 hours away from home is a lot of hours.” Or, in the words of another male employee, “The shifts are too long. And keeping in mind that in general I think women are still more responsible for running the home, it’s proven more difficult for them to adapt to that.” An ongoing discussion has existed about offering the female operators the option to job share. It was frequently mentioned in our interviews that women do not return to the plant after pregnancy because of the shifts.

Only a few interviewees mentioned that shiftwork is not good for fathers. One of the male interviewees described it this way: “I think it’s more acceptable to have the mother in the house taking care of the children in the nights while the men go out, you know, working the night shifts…It is just a culture thing.” Nevertheless, in 2011, the plant conducted a survey about employees’ preferences for 12- versus 8-h shifts. The results showed that the majority of employees, and more women than men (60% versus 50%), wanted to maintain the 12-h shifts. “This came as a shock to me,” one male worker, who thought that most resistance to the 12-h shifts came from women, told us. A female operator, a mother of three children, told us that she liked the 12-h shifts because they gave her more days off from work compared to 8-h shifts. Thus, the discourse constructing 12-h shifts as problematic for mothers was strong in the plant, even if it did not necessarily come from the mothers themselves. Despite the survey we mentioned, the plant has now changed the shift system from 12 to 8 h.

#### Too Much Physical Strain for Women

The interviews illustrate that norms of dealing with physical strain, dirt, and dust are related to what it means to be a man in the plant. Even with management support behind the 50/50 policy, more male than female managers and operators asserted that an operator’s working tasks are too difficult for women. The perception that this work is too difficult will keep the plant from reaching the 50/50 target. For example, speaking about a section of the plant where the work is considered to be dangerous and arduous, one man in administration reported: “Actually I think it is questionable whether it’s ethical to try to stuff [this section to] 50/50. I think it’s highly doubtful. And I fear that many women who have left [this section] have not done so physically unharmed.”

Some of the female employees who work outside of production, which includes the section in question, share this opinion. One of them said:

Women are not built for this work…in general, this is not a woman’s job…We women are smaller; most of us are smaller than men. The equipments (sic) are made for bigger people, and you need to use muscles that we women don’t have…That’s just biological. There is nothing we can do about it.

However, the plant redesigned the equipment to be more ergonomically appropriate for a variety of physical sizes. Nevertheless, a female supervisor who works outside of the production side said:

Personally, I think that some women are just not made for it. And a lot of women just go into [this section] and…they’re getting scared. And if you’re scared, you’re doing mistakes. And if you’re doing mistakes, there will be accidents.

However, as we pointed out before, women were seen as making fewer mistakes than men. The plant had analyzed the accident rate by gender, although management did not share the findings with us.

Despite these arguments, the female operators we interviewed who worked in this physically demanding section of production—some of them quite small, slim, and “feminine”—disagreed with the picture that was drawn of these jobs being too demanding for women. When asked which tasks were the most difficult or physically demanding, one of them said: “None of them. It can be tiring to walk long distances, but no, none of the tasks is heavy or physically demanding.” The employees also pointed out that not all men are strong: “You can find both women and men who can and cannot do this work,” one male operator told us. Another man revealed: “We guys sometimes help each other.”

The gender equality policy has been enforced at the macro level in the plant through the institutionalization of the policy itself, the establishment of a gender equality committee, official statements, public announcements, and meetings. Much less attention has been paid to how gender operates at the micro level interactionally within the plant or how traditional masculine working culture can (re)produce gender stereotypes and facilitate horizontal segregation. Employees who support the policy are not bothered by inattention to these details because, generally, they do not seem to expect any action on behalf of gender equality, other than to welcome women to the plant and ensure that the working tasks are as secure and easy as possible for both women and men. These two initiatives are seen enough to brand the plant as “modern.”

## Discussion

By examining a heavy industry plant in Iceland, which aims to create a workplace where an equal number of women and men work, we have responded to researchers who call for increased understanding of what keeps women and men employed in different fields of work, despite policy and acts supporting an integrated labor market. We show that the plant’s employees tacitly support the policy because it brands the plant as modern and the inclusion of more women is seen as enhancing the workplace environment through greater safety and care. Nevertheless, we also show a clear gap between support for the policy and doubt in its potential success, a gap that was seen by employees as more or less unproblematic and normal. Despite that, employees see gender equality as a matter of justice, whereas managers see gender equality mainly as an important business-related factor. Mangers recognize that the surrounding community will not support the existence of the plant if it does not create job opportunities for both men and women. Women’s labor force participation rate in Iceland is high (86.9% for 25–64-year-old women; OECD 2018), and the labor market where the plant is placed has been highly male-oriented. Therefore, the largest employer in the community, Metalicorp, is expected to offer employment for both genders. Thus, what appears as a progressive gender equality policy is rooted in business and local politics, which certainly is a strength for the project.

In addition to justice and business reasons for support of the policy, we found essentialist reasons among the employees. They refer to ideas about women as more capable than men are of working in a dangerous industry because women are seen as more careful and reliable by nature. Also, interviewees referred to the influence of “female” attributes on working morale and caring for others (also see Heilman and Okimoto 2007). Therefore our results support what the European Commission (2010) pointed out: Gender-based stereotypes pigeonhole women and men into different occupational roles and sectors of employment.

Yet, our analysis does not provide evidence to support studies showing that women were penalized for success in the production side of the plant, like Heilman and Okimoto’s (2007) study shows. We also saw no support for prejudice like that seen by Garcia-Retamero and López-Zafra (2006). Thus, perhaps gender equality has advanced far enough in Iceland for avoiding such outcomes, even if there were several other hindrances in Metalicorp’s project.

### Policy Versus Practice

Each time we visited the plant, the interviewed senior managers confirmed that even though the percentage of female employees was dropping, they had no intention of giving up the goal of eliminating horizontal gender segregation. Also, we did not detect decreased support among the employees, but rather we observed the opposite. Thus, even though the female employees in the production side felt socially marginalized sometimes (also see Røthing 2006), such feelings were more often addressed in our first visits than in our later ones. However, our interviews reflected what Browne (2006) saw as a disjuncture between policy and practice. This disjuncture and the fact that the gender balance decreased over the first years of the plant’s operation, and is still far from reaching the 50/50 target, has the potential to work against the project, and it serves to “prove” to uninitiated observers that gender integration ultimately does not work in practice. Thus, a failure to follow through might eventually weaken the policy itself. The discourse that “mothers don’t work long shifts” can influence the way young women see themselves as mothers and employees at the plant and, as such, reproduce stereotypical gender relations. We see this as vital because employees’ discursive options frame the potential for agency in the social context of work. Discussions that women should take “primary responsibility for raising the family” and about “gender-based characteristics” are often used for explaining why occupational segregation persists in the labor market (European Commission 2010; Heilman 2001). These discourses are represented strongly in our data, which is probably somewhat surprising in the country ranked first internationally in gender equality for a number of years running (World Economic Forum 2017) and where defamilization is strongly promoted (Bambra 2004; Eydal and Friðriksdóttir 2012; Lister 1997). Nevertheless, our data partly support the idea, presented by Ellingsæter (2013) and Jarman et al. (2012), that gender equality and essentialism work simultaneously because women were sometimes seen as better and safer operators due to the caring and prudent nature of women. Even those who doubted women’s physical capability sometimes simultaneously supported the policy because they perceived that women brought other, more positive attributes to the plant, such as caring and carefulness. However, part of the essentialism, such as the idea that the work is too physically demanding and dirty for women, also works against gender desegregation because it undermines gender inequality and reduces the likelihood of women applying for the job.

Although the management is sincere regarding the gender equality policy and the 50/50 target, our interviews with employees suggest that the existence of the policy itself is more important than its potential outcome and implementation because the policy made the plant modern. Until recently, attempts to influence a traditionally male, less-tangible working culture and transform it into a more gender-neutral, supportive culture have been minimal. The main emphasis so far has been to design all the jobs so that both genders can perform them in a safe way, advertise their gender equality policy and the 50/50 target, hold special women’s days during which women working at the plant present job opportunities to other women in the community, and have employees visit the elementary and high schools in the area to educate children about the work of the plant and to encourage both boys and girls to acquire the “right” education for the plant.

However, now the management team is aiming to do more. According to an interview with Metalicorp’s director in mass media at the end of 2017 and our recent interview with one of the middle managers, the aim for 2018 is to analyze a possible male working culture within the plant, which might make women feel uncomfortable and worth less than men. That plan is promising because gender roles produce and reproduce in interaction between women and men at work and are, as such, under the influence of the dominant working culture. However, Metalicorp does not exist in a vacuum, and the gender roles outside the plant also matter. Most of those who live outside Metalicorp’s surrounding community apply for employment at the plant through a recruitment agency. Remarkably, our interview with a representative from the agency suggested very little understanding of Metalicorp’s 50/50 target and demonstrated stereotypical gendered understandings of women’s and men’s job skills and gender roles. This unawareness was disappointing and showed that the plant’s gender equality strategy had not been integrated well enough into the recruitment process.

### Strengths, Limitations, and Future Research Directions

Key strengths of our study are the unusual context in which it has been conducted—a relatively new, hazardous heavy industry plant where, from the beginning, the management publically declared war on gender segregation—and our ability to follow the gender equality project and its discourse from its early days. The plant is rather “closed” due to security reasons, but nevertheless, the management team trusted us to observe the different sites of the plant, dressed in overalls with helmets and wearing security shoes just like every employee. In the most dangerous divisions, we were accompanied by a representative from the management team. That partly restricted our observations and made us more reliant on interviewing; at the same time, it made the observation fruitful because we were able to ask and discuss what we saw with the one who accompanied us.

Our own gender (both authors are women) may have inhibited how interviewees spoke with us about the gender equality policy. Nevertheless, we have been pleased with the interviewees’ openness on a variety of topics relating to gender, and responses to our questions do not suggest such inhibition. Our use of snowball sampling may have oriented our sample toward the extremes—those who are particularly supportive or particularly unsupportive of gender equality efforts; however, again, the range of our interviews does not bear this dichotomy out. We have drawn conclusions about why women left Metalicorp based on interviews with current and former employees. To strengthen the conclusions about persisting gender desegregation and uneven gender ratios, it is our hope that future research will include more interviews with those who left the plant for different reasons and with those who are not willing to apply for jobs at Metalicorp despite living in the nearby community.

The managers at Metalicorp are still striving to find ways to reach the 50/50 target. Their next step is to focus on the possible male working culture within the plant and the psychosocial working environment, that is, the ways in which the gender ideology is reproduced at the organizational level and in the gender relations. The plant also is working toward compliance with Iceland’s gender equality laws, which address the importance of changing traditional gender images and work against negative stereotypes regarding the roles of women and men. In that light, we look forward to following the progress of the 50/50 target and to seeing whether this new focus will bring the plant closer to the aim of hiring an equal percentage of women and men to the plant.

### Practice Implications

Metalicorp’s project has created a unique opportunity for the understanding of progress and barriers to gender desegregation at work. Our findings show, for instance, that what keeps women away from such jobs is persistent ideas about stereotypical gender roles and the gap between the gender equality policy and the expectations employees have about the actual usefulness of the policy. Therefore, it will be of interest to follow this year’s project, aiming to work in a more focused manner with the gender-related working culture and possible pressure of exclusion as a result of being the minority gender within Metalicorp.

Our results are also of academic interest because they can influence the theoretical discussion about hindrances and success in eliminating horizontal gender segregation at work. In addition, they can provide professionals, such as policymakers and organizational administrators, with concrete ideas about how to work toward gender desegregation in other, and even less hazardous, workplaces, as well as what to avoid.

### Conclusions

Laws, core values, and gender equality strategies are clear prerequisites for horizontal gender desegregation and the policy to hire an equal number of women and men to a hazardous workplace such as Metalicorp. Also, management’s support of the policy is vital. This is also what Metalicorp has been doing well. However, to fully be able to successfully address gender segregation, actions must also be taken to improve the psychosocial working environment and female-friendly working culture, aiming to go beyond common discourses about gender stereotypes and, at the same time, recognizing that women’s and men’s needs can be different. That can sound like a contradiction, but that does not need to be the case. Because targeting the masculine working culture is what the management has decided to do, we look forward to following the progress of the project. Whether the new strategy will bring the plant closer to the 50/50 target, despite the fact that part of the reason why more women have not been hired to the plant lies in gender stereotypes and gender roles outside the plant. This is worth continued research.
